# Gd-DTPA Adsorption on Chitosan/Magnetite Nanocomposites

**DOI:** 10.1186/s11671-016-1363-3

**Published:** 2016-03-31

**Authors:** Ie. V. Pylypchuk, D. Kołodyńska, M. Kozioł, P. P. Gorbyk

**Affiliations:** Nanomaterials Department, Chuiko Institute of Surface Chemistry of the National Academy of Sciences of Ukraine, 17 General Naumov Str., 03164 Kyiv, Ukraine; Department of Inorganic Chemistry, Faculty of Chemistry, Maria Curie Skłodowska University, M. Curie Skłodowska Sq. 2, 20-031 Lublin, Poland

**Keywords:** Magnetite, Gd-DTPA adsorption, Chitosan, Neutron capture therapy, Hybrid nanocomposites

## Abstract

The synthesis of the chitosan/magnetite nanocomposites is presented. Composites were prepared by co-precipitation of iron(II) and iron(III) salts by aqueous ammonia in the 0.1 % chitosan solution. It was shown that magnetite synthesis in the chitosan medium does not affect the magnetite crystal structure. The thermal analysis data showed 4.6 % of mass concentration of chitosan in the hybrid chitosan/magnetite composite. In the concentration range of initial Gd-DTPA solution up to 0.4 mmol/L, addition of chitosan to magnetite increases the adsorption capacity and affinity to Gd-DTPA complex. The Langmuir and Freundlich adsorption models were applied to describe adsorption processes. Nanocomposites were characterized by scanning electron microscopy (SEM), differential thermal analysis (DTA), Fourier transform infrared spectroscopy (FTIR), X-ray diffraction (XRD), and specific surface area determination (ASAP) methods.

## Background

Increasing interest in multifunctional nanomaterials for biomedical application necessitates detailed investigations of processes occurring at the nanolevel. Development of such nanomaterials brings scientists closer to realization of nanorobot concept—targeted drug delivery, cell recognition, and complex therapy and diagnostics. Creation of hybrid biopolymer/mineral nanomaterials can lead to development of novel nanocomposites that are sensitive to pH, temperature, magnetic field, and other physicochemical actions [1].

Nanocomposites based on magnetite (Fe_3_O_4_) are widely used for magnetic resonance imaging (MRI) and targeted drug delivery [2–5]. Promising trends of using magnetic materials with advanced surface are preparation of magnetosensitive nanocomposites with natural biopolymers (e.g., chitosan). Combination of chitosan and magnetite properties opens the way to creation of Pylypchuknew effective pH-controllable drug delivery and release systems with high biocompatibility. Chitosan-inorganic mineral composites have attracted researchers’ attention due to their good adsorption properties, high speed of adsorption kinetics, and some technical advantages to operate with them. The previous studies exhibit great potential of chitosan-inorganic mineral composites [1–6].

There are a number of articles reporting on synthesis and properties of magnetite/chitosan nanomaterials. For instance, hydrogel, chitosan (CS) cross-linked carboxymethyl-β-cyclodextrin (CM-β-CD) polymer modified Fe_3_O_4_ magnetic nanoparticles were synthesized in [7]. Magnetic chitosan beads were synthesized by incorporating *N*,*O*-carboxymethyl chitosan-coated magnetic nanoparticles (NOCC-MNPs) into chitosan-citrate gel beads (CCGBs) for adsorbing Cu(II) ions. The maximal adsorption capacity as estimated by the Langmuir model was 294.11 mg/g [7]. A magnetic composite material composed of nanomagnetite (NMT), heulandite (HE), and cross-linked chitosan was prepared and used as an adsorbent for methylene blue (MB) and methyl orange (MO). The adsorption of MB and MO followed the pseudo-second-order kinetics, and the maximum adsorption capacities were 45.1 and 149.2 mg/g at pH 5.5, respectively [8]. The authors developed a novel chitosan/Al_2_O_3_/magnetic iron oxide nanoparticle composite acting as an adsorbent for removing MO, a model anionic dye, from aqueous solution. The adsorption isotherm was well described by the Langmuir model and showed a high MO adsorption capacity (1.27 mmol/g, i.e., 417 mg/g at 25 °C) [9].

Biofunctionalized chitosan@Fe_3_O_4_ nanoparticles were synthesized by combining Fe_3_O_4_ and CS chemically modified with PEG and lactobionic acid in one step [10]. A novel pyridinium-diethylenetriamine magnetic chitosan (PDFMC) was prepared and used for magnetic separation of Cr(VI) from aqueous solution. The PDFMC worked well on removal of Cr(VI) in any condition of acidic, neutral, and basic solutions with the capacity (*q*_max_) of 176 mg/L (at pH 3, acidic), 124 mg/L (pH 6, near-neutral), and 86 mg/L (pH 9, basic) based on the Langmuir isotherm model [11]. Cellulose grafted to nanomagnetites was found to be an efficient biopolymer composite for catalysis of Friedel-Crafts reaction between isatins and indoles, leading to selective synthesis of 3-hydroxy-3- indolylindolin-2-ones [12]. As another example, magnetic nanoparticles double-coated with different concentrations of dextran sulfate or reduced dextran and chitosan solutions were formed by layer-by-layer deposition. Chitosan-coated magnetic nanoparticles have been synthesized and developed as a highly efficient nano-adsorbent for the removal of Hg^2+^ ions from industrial aqueous and oily samples. The results confirmed formation of narrow-dispersed nanoparticles with a mean average diameter of about 10 nm [13].

Chitosan microspheres are the most widely studied drug delivery systems for the controlled release of drugs, such as antibiotics, antihypertensive agents, anticancer agents, proteins, peptide drugs, and vaccines [14]. In this respect, composites containing chitosan are widely used as a drug carrier for gadolinium and its biocompatible complexes (e.g., Gd-diethylenetriaminepentaacetic (DTPA), Gd-DOTA). Such substances find application as contrast agent in MRI due to paramagnetic properties of Gd^3+^ [15]. The conjugates of the complexes of Gd-DTPA with low-molecular-weight chitosan are described in [16–22]. In addition, gadolinium, due to its nucleus property to capture thermal neutron with release of γ-quantum’s and Auger electrons, can be used in the neutron capture therapy (NCT) of cancer [22–31].

Unfortunately, there is no relevant information about Gd-DTPA adsorption on magnetite-chitosan composites. The main goal of this article is to obtain magnetite-chitosan nanocomposites and obtain information with respect to the Gd-DTPA adsorption on these materials.

Development of hybrid magnetic chitosan nanocomposites and peculiarities of Gd-DTPA adsorption on these nanocomposites is an important task for further development of composites for biomedical destination with a wide range of functions—targeted drug delivery, pH-controllable release, and chemo-, immuno-, and radiotherapy as well as diagnostic agents.

## Experimental Part

### Materials

All reagents were of analytical grade and used without further purification. Demineralized water was used for preparation of all sample solutions (Hydrolab).

*Chitosan*, Sigma-Aldrich, with a molecular weight from 190,000 to 370,000 Da, degree of deacetylation not less than 75 %, and solubility of 10 mg/mL.

*Gd-DTPA* was prepared by dissolution of two equivalents of Gd_2_O_3_ in 0.04 M DTPA to obtain 0.04 M Gd-DTPA solution. Obtained solution was adjusted to pH = 7.26 by NaOH.

## Methods

### Differential Thermal Analysis

Thermal behavior of magnetite and its nanocomposites was determined by thermogravimetric analysis (TGA) using Q50 TGA instrument. TGA measurements of 4–25-mg samples were carried out at 10 °C/min heating rate in the range of 25–1000 °C under nitrogen atmosphere with a flow rate of 50 cm^3^/min.

### Surface Area and Average Pore Diameter (ASAP) Measurements

Specific surface areas and pore volumes were determined from the low-temperature nitrogen adsorption data (automatic sorption analyzer ASAP 2020, Micromeritics, USA). Before measurements, the samples were outgassed at 60 °C.

### Carbon, Hydrogen, and Nitrogen Analysis

Elemental analysis of chitosan-silica composite was carried out by using the CHN/O analyzer (Perkin Elmer, Series II CHNS/O Analyzer 2400). The analysis was made at the combustion temperature of 925 °C and the reduction temperature of 640 °C.

### Surface Morphology Analysis

The surface morphology of chitosan-magnetite composite was observed by using a scanning electron microscope (SEM, LEO 1430VP, Carl Zeiss, Germany).

### The Fourier Transform Infrared Spectra

The Fourier transform infrared spectra were registered using a Cary 630 ATR-FTIR instrument (Agilent Technologies) by the attenuated total internal reflection technique.

### The pH of the Point of Zero Charge pH_pzc_

The pH of the point of zero charge pH_pzc_ was measured using the pH drift method. The pH of the sorbent in the 0.01 M NaCl solution was adjusted between 2 and 12 by adding 0.01 M NaOH and 0.01 M HCl. To 50 cm^3^ of the solution, 0.2 g of the adsorbent was added, and after 24 h, the final pH was measured.

### Magnetite Synthesis

In 1 L of deionized water, 24 g of ferrous chloride (FeCl_2_) and 48 g of Ferric chloride solution (FeCl_3_) were dissolved. This solution was added dropwise to 250 mL of ammonia solution (NH_4_OH, 25 % in water). Black precipitate was collected and washed several times by distilled water to pH = 7.

The synthesis of magnetite was carried out by the co-precipitation of iron salts according to the reaction

$$ {\mathrm{Fe}}^{+2} + 2{\mathrm{Fe}}^{+3} + 8{\mathrm{NH}}_4\mathrm{O}\mathrm{H}\ \to\ {\mathrm{Fe}}_3{\mathrm{O}}_4 + 4{\mathrm{H}}_2\mathrm{O} + {{8\mathrm{N}\mathrm{H}}_4}^{+} $$

### Chitosan/Magnetite Nanocomposite Synthesis

**Fig. 1:**
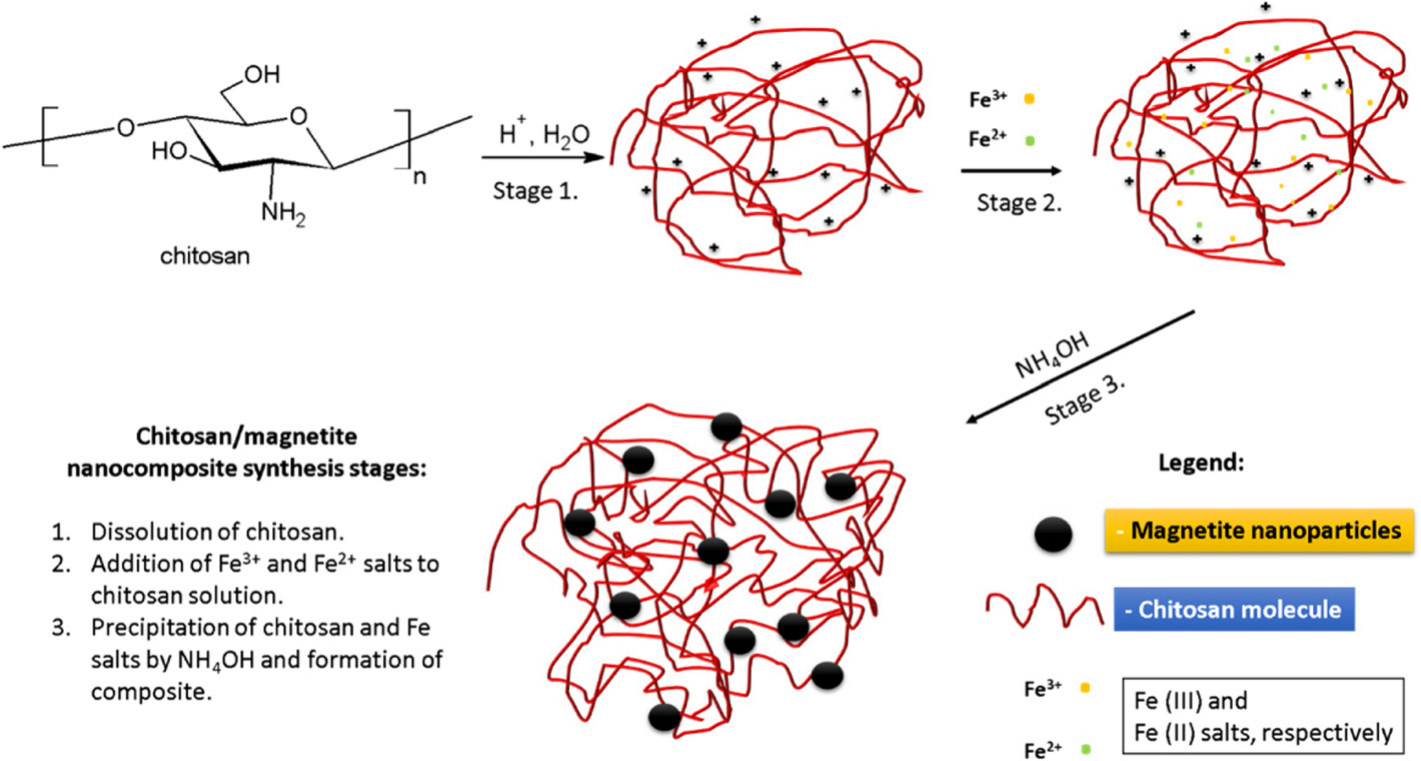
Scheme of chitosan/magnetite nanocomposite synthesis

Chitosan/magnetite nanocomposites were made by co-precipitation method. Chitosan solution was obtained by dissolving 0.5 g of chitosan (low Mw) in 50 cm^3^ of 1 % CH_3_COOH. The obtained solution was mixed with 0.5 L solution of Fe^2+^ and Fe^3+^ (24 g FeCl_3_·6H_2_O + FeSO_4_·2H_2_O) and left stirring overnight at 40 °C. The resulting solution was added dropwise to 150 mL of 25 % NH_4_OH solution in water (scheme in Fig. 1). The precipitate was collected by a permanent magnet and washed by doubly distilled water several times up to pH = 7. The obtained composite was dried in air at 60 °C. Ten grams of black powder was obtained.

## Results and Discussion

There are many ways to obtain chitosan-magnetite nanocomposites. For instance, magnetic nanoparticles with an average crystallite size of 21.8 nm were covered in a core/shell type; magnetite/silica, magnetite/chitosan, and a double-shell magnetite/silica/chitosan were developed for attaching an antineoplastic drug [32]. Chitosan-coated magnetite nanocomposites (Fe_3_O_4_/CS) were prepared under different external magnetic fields by the co-precipitation method [33]. A chitosan-based hydrogel, graft-copolymerized with methylenebisacrylamide and poly(acrylic acid) (i.e., CS-co-MMB-co-PAA), was employed in the studies on the adsorption kinetics of Pb(II), Cd(II), and Cu(II) ions in aqueous solution [34].

Various magnetic films of chitosan and the synthesized magnetite nanopowders containing different concentrations of the latter were prepared by the ultrasonication route [35].

The use of the biopolymer chitosan as a template for the preparation of magnetite and magnetite/silver core/shell nanoparticle systems, following a two-step procedure of magnetite nanoparticles in situ precipitation and subsequent silver ion reduction, is discussed in [36].

A magnetic nanoparticle drug carrier for the controlled drug release that responds to the change in external temperature or pH was described in [37], with characteristics of longer circulation time and reduced side effects. The novel nanocarrier is characterized by a functionalized magnetite (Fe_3_O_4_) core that is conjugated with drug via acid-labile hydrazone bond and encapsulated by the thermosensitive smart polymer, chitosan-g-poly(*N*-isopropylacrylamide-co-*N*,*N*-dimethylacrylamide) [chitosan-g-poly(NIPAAm-co-DMAAm)]. The polyelectrolyte complex (PEC) effect between hyaluronic acid (HA) and chitosan was explored to recover HA from the fermentation broth. Chitosan was conjugated with the magnetic nanoparticles by the co-precipitation method to facilitate its recovery [38]. Chitosan/magnetite nanocomposite was synthesized induced by magnetic field via in situ hybridization under ambient conditions. The saturated magnetization (Ms) of nanomagnetite in chitosan was 50.54 emu/g, which is as high as 54 % of bulk magnetite [39].

In this work, we used a method based on co-precipitation of Fe^2+^/Fe^3+^ salts by aqueous ammonia in 0.1 % chitosan solution.

It is well known that chitosan is soluble in water in acidic media (pH = 2–6). At this pH, chitosan swells and its chains undergo deploying due to electrostatic repulsion of positively charged –NH_3_^+^ groups.

Iron salts, after mixing with chitosan solution, surrounding the chitosan molecule form complexes with its amino groups [40]. The addition of ammonia affects the charge of chitosan molecule, resulting in shrinkage of chitosan and precipitation of Fe_3_O_4_, and leads to the formation of chitosan-magnetite aggregates. The scheme of synthesis is presented in Fig. 1.

### SEM

**Fig. 2:**
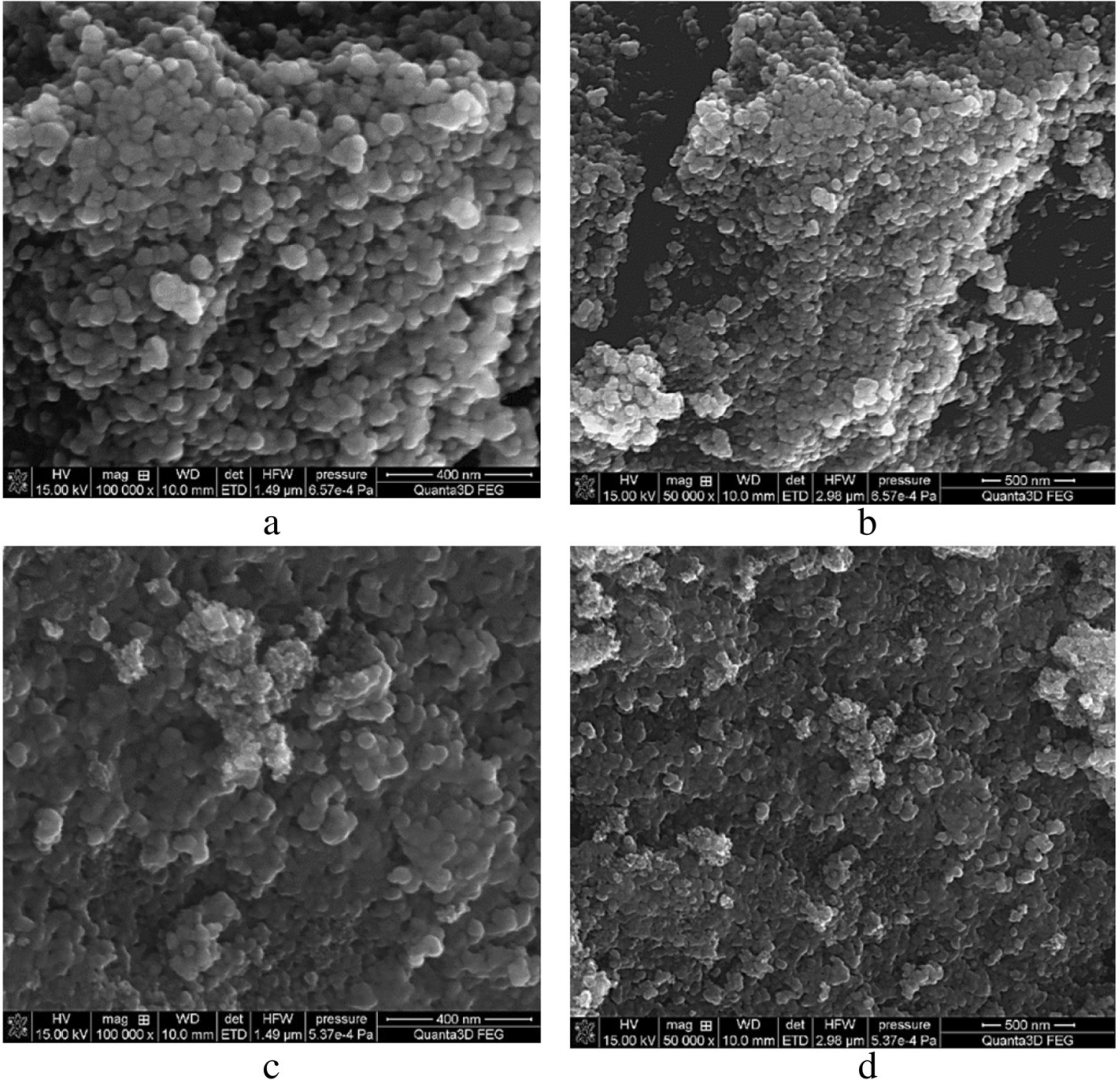
SEM images of magnetite (**a**, **b**) and magnetite/chitosan composites (**c**, **d**)

The obtained magnetite and composite powders were analyzed by SEM. In the SEM images of magnetite particles (Fig. 2a, b), we can observe that they are of spherical shape and their size varies from 20 to 40 nm. Nanocrystals form aggregates up to 200 nm, which can assemble in large aggregates. The specific surface area, calculated by the BET method, is equal to 132 m^2^/g.

Chitosan/magnetite nanocomposite (Fig. 2c, d) has similar but not identical surface with some differences. Chitosan/magnetite nanocomposite has a rough and irregular surface, which is common for hybrid materials. Compared to unmodified magnetite (Fig. 2a, b), composite particles surface and pores between them look like filled by polymer species. Filling of magnetite particle aggregates with polymer causes decrease in the specific surface area of composite (101 m^2^/g). The influence of chitosan addition to magnetite on the specific surface area of composite is discussed further.

### Specific Surface Area Determination

The isotherm plots were used to calculate the specific surface area and the average pore diameter of the magnetite and chitosan/magnetite nanocomposite.

**Fig. 3:**
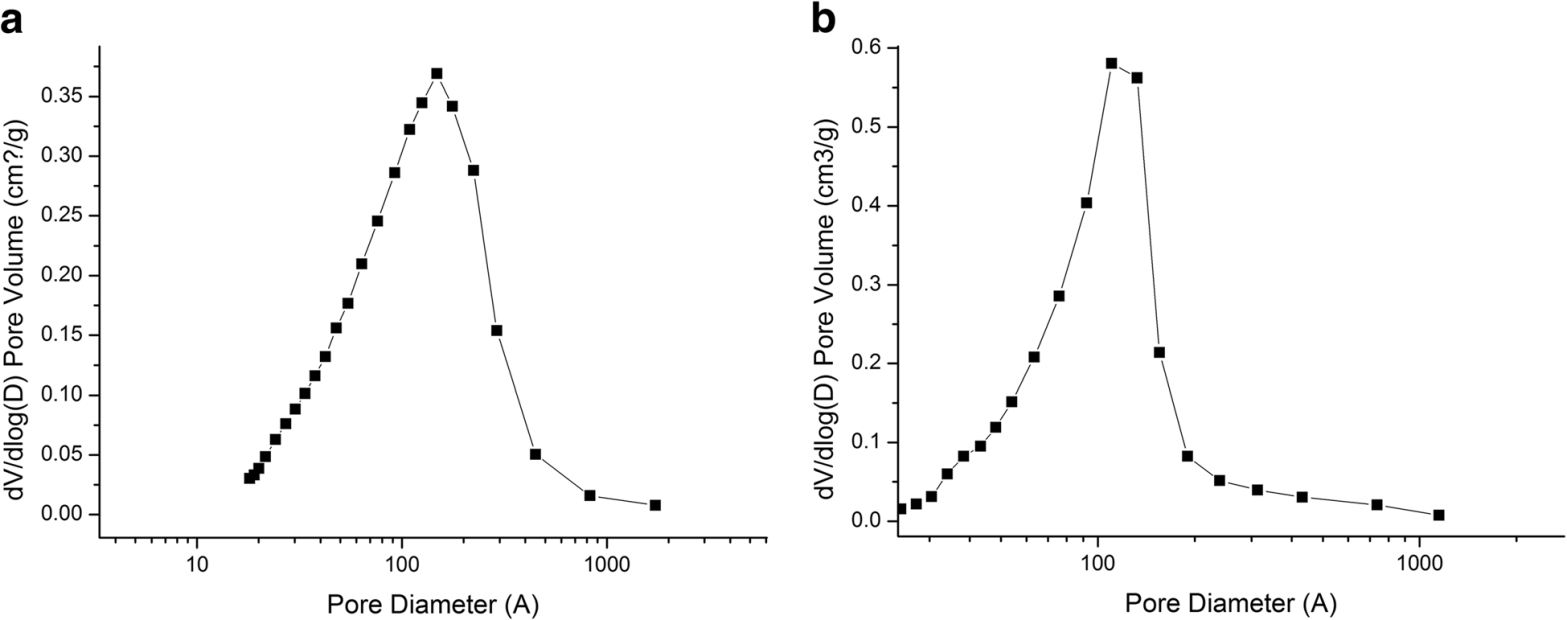
Pore size distribution for magnetite (**a**) and magnetite/chitosan composites (**b**) calculated by the BJH analysis method

The pore size and volume analysis was made using two models. The first model is based on the Barrett-Joyner-Halenda (BJH) method implemented in the firm software. The second one takes into account slit/cylindrical pores and voids between spherical particles with the self-consistent regularization (SCV/SCR) [41–44]. According to the calculations based on the BJH method (Fig. 3a, b), magnetite mesopores of 10-nm size prevail. In the case of magnetite/chitosan composites, it can be equal to magnetite.

According to the results of ASAP analysis of the chitosan/magnetite composites compared to unmodified magnetite, the BET surface area of composite decreased after synthesis of magnetite in the chitosan medium (Table 1).

**Table 1:** The BET and the Langmuir surface areas of Fe_3_O_4_ and Chitosan/Fe_3_O_4_ composites

Material	BET surface area	Langmuir surface area
Fe_3_O_4_	134.0 m^2^/g	195.7 m^2^/g
Chitosan/Fe_3_O_4_	101.2 m^2^/g	148.7 m^2^/g

**Fig. 4:**
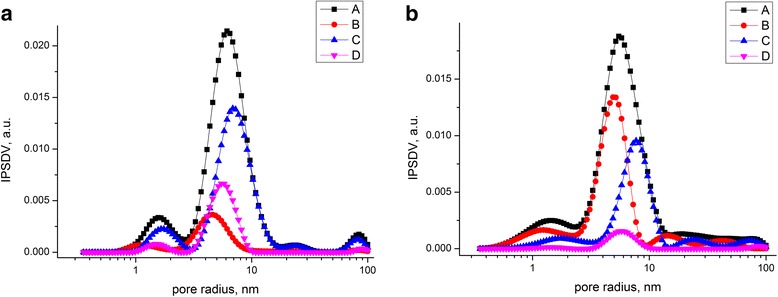
Pore size distribution for magnetite (**a**) and magnetite/chitosan composites (**b**) calculated by the SCV/SCR analysis method (*А*—summ of pore impact, *B*—slit-shaped pores, *C*—cylindrical pores, *D*—voids between spherical particles)

Pore size distribution calculated using the SCV/SCR procedure (integral adsorption equations based on a complex model with slit-shaped and cylindrical pores and voids between spherical nonporous particles packed in random aggregates) is presented in Fig. 4. It was found that all obtained samples of chitosan-magnetite composite have an average pore diameter up to 10 nm and can be defined as mesopores. The presence of mesopores for all obtained samples of the composites was confirmed by the diagrams of pore size distribution (Fig. 4), which was obtained by the adsorption branch of the isotherm using the SCV/SCR method.

For magnetite, the contribution of cylindrical pores calculated by the SCV/SCR method gives 62 %, contribution of pores between particles is 22 %, and slit-like pores 15.9 %. For chitosan/magnetite nanocomposite, contribution of cylindrical pores is equal to 56.3 %, contribution of pores between particles is 38.47 %, and slit-like pores 5.2 %. Changes in pore distribution can be caused by polymer filling in the cylindrical pores and redistribution in favor of the pores between particles.

### FTIR Analysis

**Fig. 5:**
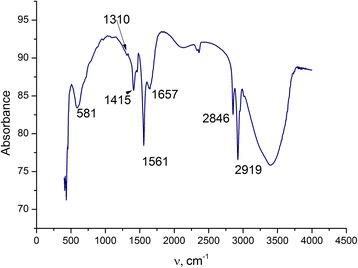
FTIR spectra of chitosan/magnetite composite

FTIR spectrum confirms the presence of chitosan in composite and is presented in Fig. 5.

In the FTIR spectrum of chitosan/magnetite, the broad adsorption band from 3600 to 3100 cm^−1^ corresponds to the stretching vibrations O–H of hydroxyl groups. The bands at 2919 and 2846 cm^−1^ were attributed to the asymmetric and symmetric CH_2_ stretch vibrations of chitosan, respectively. The AB at 1657 cm^−1^ can be coupled with water molecules. The band at 1561 cm^−1^ corresponds to the deformation vibrations of -NH_2_; 1415 and 1310 сm^−1^ for C–H bending vibrations, 1310 сm^−1^ for asymmetric С–О–С stretching vibrations, and 1080 сm^−1^ for С–О stretching vibration of СН–ОН were observed. Magnetite adsorption bands can be observed at 581 cm^−1^.

### TGA

**Fig. 6:**
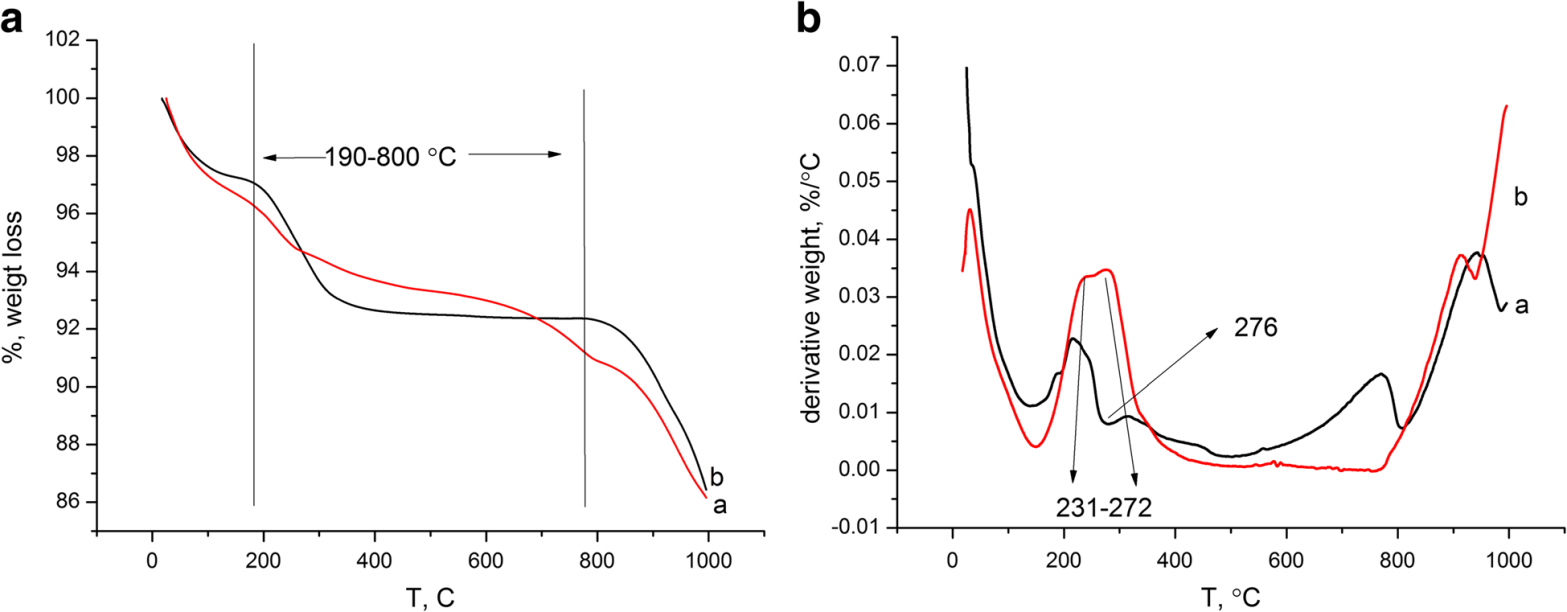
Thermograms of magnetite (**a**) and magnetite/chitosan (**b**)

The thermal stability of the magnetite and magnetite/chitosan composite is presented in Fig. 6a, b, respectively.

The low temperature weight loss from 25 up to 190 °C (endothermic peak) in the magnetite and hybrid composite corresponds to evaporation of physically adsorbed water. Chitosan started to undergo thermal destruction at 190 °C. The temperature range from 227 to 286 °C (exothermic process) could be assigned to chitosan oxidation (oxidation of –NH_2_ and –CH_2_OH groups).

Decomposition of the polymer chain in magnetite/chitosan starts from 190 °C and practically is over at 410–420 °C. In the temperature range from 289 to 775 °C, we can observe descending endothermic curve which can be explained by chitosan destruction (decarboxylation of oxidized –CH_2_OH groups, macromolecule chain breaks, release of nitrogen oxides from oxidized amino groups, etc.). In the case of chitosan/magnetite, the onset of the chitosan decomposition shifted to the temperatures of up to 20 °C.

The weight loss at the 190–800 °C temperature range for magnetite/chitosan composite was 4.6 % [45].

### Elemental Analysis

For the obtained composites, the total carbon content (CHN elemental analysis) as well as carbon concentration on the composite surface (EDAX analysis) was investigated (Table 2). As expected, carbon concentration on the surface of composite is higher than the total C content. The CHN elemental analysis data is in good agreement with the chitosan content in the composite obtained by the thermogravimetric analysis.

**Table 2:** Comparison of the chitosan/magnetite and magnetite element composition

Chitosan/magnetite composite			Magnetite		
Element	Wt., %	At., %	Element	Wt., %	At., %
CHN analysis					
C	2.28	–	C	–	–
N	0.52	–	N	–	–
H	0.45	–	H	–	–
EDAX of material surface					
C	15.33		C	8.73	19.09
Fe	27.71	9.49	Fe	56.31	26.49
O	52.99	63.33	O	31.79	52.19

### XRD Analysis

**Fig. 7:**
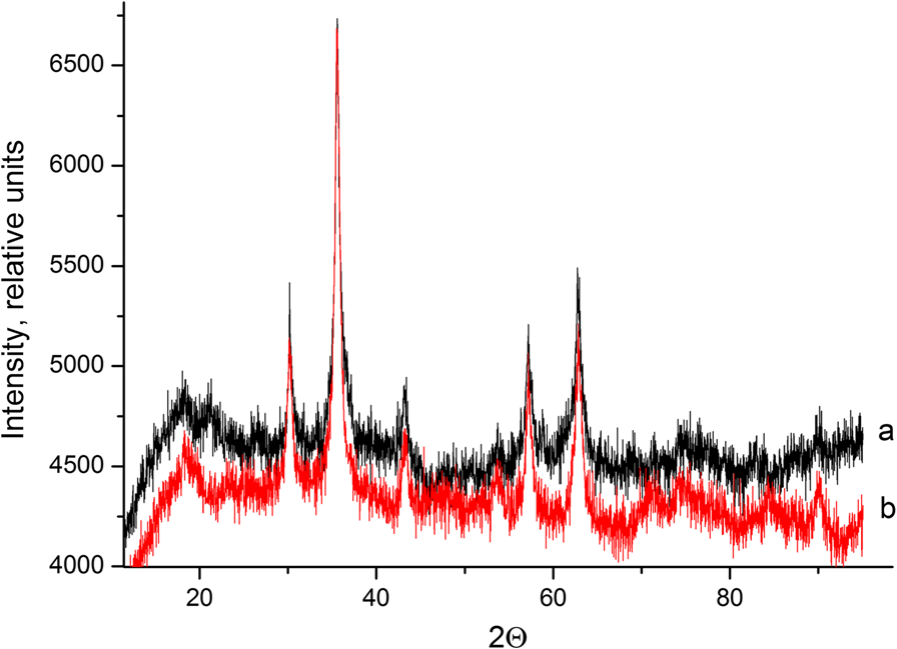
XRD patterns for magnetite (curve *a*), and magnetite/chitosan composites (curve *b*)

According to the XRD analysis, the main phase in both magnetite and chitosan/composite is iron oxide Fe_3_O_4_ (Fig. 7). No difference in crystal structure was observed.

### Adsorption of Gd-DTPA

Adsorption of Gd-DTPA ions on the surface of nanocomposite was calculated by the formula *q*_e_ = (*С*_0_ − *С*_eq_)*V*/*m*, where *C* and *C*_eq_ are the initial and equilibrium concentration of ions in solution, respectively, mg/mL; *V* is the volume of solution (cm^3^); and *m* is the mass of adsorbent (g).

**Fig. 8:**
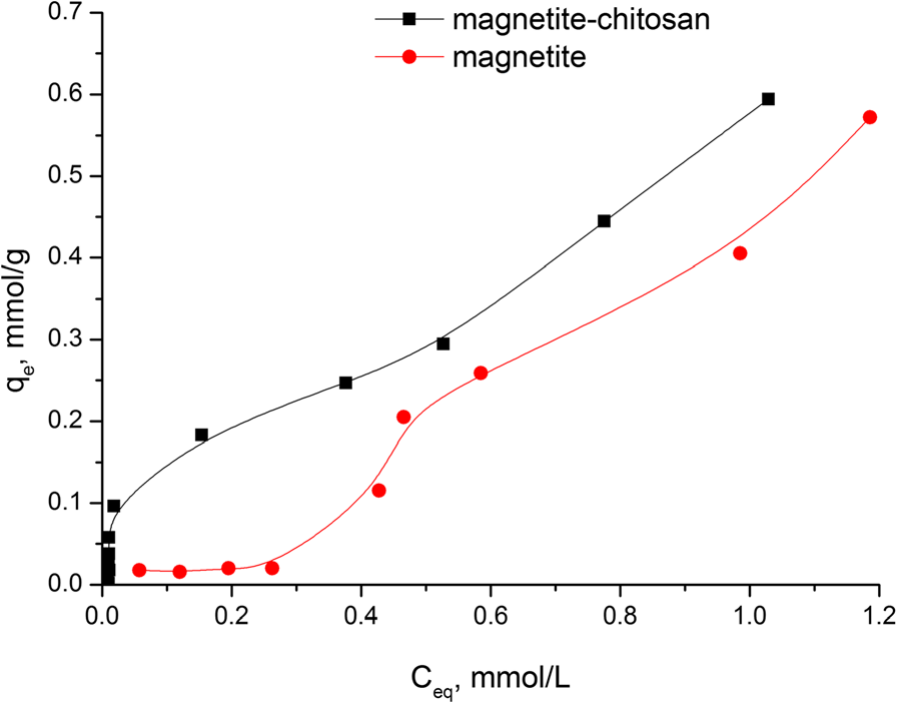
Adsorption isotherm of Gd-DTPA complex on the magnetite and chitosan-magnetite composite (pH 7.23)

Adsorption isotherm of Gd-DTPA complex on the magnetite and chitosan-magnetite composite at point of zero charge (pH 7.23) is presented in Fig. 8.

Surface adsorption on chitosan-magnetite composite is due to the presence of Fe–OH groups at the surface of iron oxides. These groups attain negative or positive charge by dissociation

1 $$ \equiv \mathrm{FeOH}\to \equiv {\mathrm{FeO}}^{-}+{\mathrm{H}}^{+} $$

or association of protons

2 $$ \equiv \mathrm{FeOH}+{\mathrm{H}}^{+}\to {{\mathrm{FeOH}}_2}^{+} $$

Therefore, the surface charging is a pH dependent. From the literature, it is well known that the pzc will vary with the particle concentration, the ionic strength of the medium. The magnetite surface is positively charged up to pH ≈ 6.8. Therefore, at decreased pH, the negatively charged Gd-DTPA complexes can be adsorbed. At higher pH, their repulsion from the negative sites at the magnetite surface reduces the adsorbed amount.

The Langmuir and Freundlich isotherm models were applied to obtain data of Gd-DTPA adsorption mechanism on nanocomposite surface.

The Langmuir model is based on the assumption that maximum adsorption occurs when saturated monolayer of solute molecules is present on the adsorbent surface and the energy of adsorption is constant and there is no migration of adsorbate molecules in the surface plane [45].

The essential characteristic of Langmuir isotherm can be expressed by the *R*_L_ parameter, which determines the favorable conditions for isotherm. If *R*_L_ < 1, the process is favorable, if *R*_L_ = 1, linear, and if *R*_L_ > 1, unfavorable. As we can see, the adsorption process of Gd-DTPA on the chitosan/magnetite composite surface is favorable [46].

The linearization for Freundlich equation is empirical and applicable to adsorption on heterogeneous surfaces as well as multilayer adsorption. As seen from Table 3, Freundlich isotherm correlation coefficient *R*^2^ = 0.837 that confirms appropriate fitting to that model. According to that model, the maximal adsorption capacity of Gd-DTPA complex on magnetite/chitosan composite is 0.59 mmol/g.

**Table 3:** Langmuir and Freundlich parameters for adsorption of Gd-DTPA complex on the magnetite and chitosan-magnetite composite

		Langmuir model				Freundlich model		
	*q*_e_ exp, mmol/g	*q*_0_	*K*_L_	*R*_L_	*R* ^2^	*K*_F_	1/*n*	*R* ^2^
Magnetite	0.571965	0.175349	0.723	0.0269	0.30050	2.67	0.724	0.83700
Magnetite/chitosan composite	0.594240	1.258178	0.734	0.0265	0.04390	1.74	1.353	0.72960

In the concentration range from 0.4 to 1.2 mmol/L, the adsorption capacity of composite is not much more higher than for pure magnetite. In the concentration range of the initial solution up to 0.4 mmol/L, addition of chitosan increased the adsorption capacity and affinity to Gd-DTPA complex. This parameter is very important for loading of Gd-DTPA micro quantities for possible use in medicine.

Unfortunately, there is no relevant information about Gd-DTPA adsorption on magnetite-chitosan composites. Adsorption capacities of the obtained magnetite-chitosan composites can be compared with other magnetite-based composites. For example, composite adsorbent prepared by entrapping cross-linked chitosan and nanomagnetite on the heulandite surface was used to remove Cu(II) and As(V) in aqueous solution. The composite gave the maximum equilibrium uptake of Cu(II) and As(V) of 17.2 (0.26875 mmol/g) and 5.9 mg/g (0.07878 mmol/g) in the initial concentration ranges of 16–656 and 17–336 mg/L, respectively [47]. In [48], chitosan/magnetite nanocomposite beads were prepared by a simple and effective process. The maximum adsorption capacities for Pb(II) and Ni(II), which occurred at pH 6 at room temperature, were as high as 63.33 (0.30564 mmol/g) and 52.55 mg/g (0.8952 mmol/g), respectively, according to the Langmuir isotherm model [48].

A comparison of the results obtained in this article (0.6 mmol of Gd-DTPA per gram of composite) with other authors leads to the conclusion about the prospects of the obtained nanocomposites for adsorption of Gd-DTPA complexes.

## Conclusions

The synthesis of chitosan/magnetite nanocomposites was made. It was shown that the magnetite synthesis in the chitosan medium does not affect magnetite crystal structure. The TGA data showed 4.6 % of mass concentration of chitosan in hybrid chitosan/magnetite. Despite low chitosan content, the adsorption properties of hybrid chitosan/magnetite composite with respect to Gd-DTPA increased in the micro quantity region to those of magnetite. In general, increase of Gd-DTPA adsorption in chitosan/magnetite composite, compared to unmodified magnetite, can be explained by the advent of chitosan. Interaction between chitosan and Gd-DTPA molecule can be connected with organic nature of these molecules coupled with electrostatic interaction. Good correlation with the Freundlich adsorption model can be explained by hybrid (organo-inorganic) nature of adsorbent.
